# Culture adaptation for enhanced biogas production from birch wood applying stable carbon isotope analysis to monitor changes in the microbial community

**DOI:** 10.1186/s13068-023-02328-w

**Published:** 2023-05-06

**Authors:** Seyedbehnam Hashemi, Linn Solli, Kristian M. Lien, Jacob J. Lamb, Svein Jarle Horn

**Affiliations:** 1 Department of Energy and Process Engineering, Norwegian University of Science and Technology (NTNU), 7034 Trondheim, Norway; 2 Norwegian Institute of Bioeconomy Research (NIBIO), 1433 Ås, Norway; 3 Faculty of Chemistry, Biotechnology, and Food Science, Norwegian University of Life Sciences (NMBU), 1432 Ås, Norway

**Keywords:** Biogas, Carbone isotope analysis, Lignocellulose, Microbial community, Steam explosion

## Abstract

Birch wood is a potential feedstock for biogas production in Northern Europe; however, the lignocellulosic matrix is recalcitrant preventing efficient conversion to methane. To improve digestibility, birch wood was thermally pre-treated using steam explosion at 220 °C for 10 min. The steam-exploded birch wood (SEBW) was co-digested with cow manure for a period of 120 days in continuously fed CSTRs where the microbial community adapted to the SEBW feedstock. Changes in the microbial community were tracked by stable carbon isotopes- and 16S r RNA analyses. The results showed that the adapted microbial culture could increase methane production up to 365 mL/g VS day, which is higher than previously reported methane production from pre-treated SEBW. This study also revealed that the microbial adaptation significantly increased the tolerance of the microbial community against the inhibitors furfural and HMF which were formed during pre-treatment of birch. The results of the microbial analysis indicated that the relative amount of cellulosic hydrolytic microorganisms (e.g. *Actinobacteriota* and *Fibrobacterota*) increased and replaced syntrophic acetate bacteria (e.g. *Cloacimonadota*, *Dethiobacteraceae*, and *Syntrophomonadaceae*) as a function of time. Moreover, the stable carbon isotope analysis indicated that the acetoclastic pathway became the main route for methane production after long-term adaptation. The shift in methane production pathway and change in microbial community shows that for anaerobic digestion of SEBW, the hydrolysis step is important. Although acetoclastic methanogens became dominant after 120 days, a potential route for methane production could also be a direct electron transfer among *Sedimentibacter* and methanogen archaea.

**Supplementary Information:**

The online version contains supplementary material available at 10.1186/s13068-023-02328-w.

## Introduction

Preventing climate change requires a transition from use of fossil fuels to renewable energy sources in order to reduce greenhouse gas emissions. Such a shift to renewables should happen before reaching an irreversible point in global warming [1]. Renewable energy is more sustainable than finite fossil fuel resources. Among all sources of renewable energies, biomass utilization for bioenergy production (including biogas) can be a carbon-neutral process with a minimum contribution to global warming and even carbon-negative if merged with a carbon capture and storage system [2, 3]. Lignocellulosic materials account for approximately 50% of the biomass in the world (about 181.5 billion tons per year) making them an abundant source for biogas production [4, 5]. Generally, lignocellulosic materials consist of cellulose (up to 55%), hemicellulose (up to 35) and lignin (up to 40%) [6]. Even though lignocellulosic materials are an available source for biogas production, these biomasses are generally recalcitrant and difficult to degrade by enzymatic activities of microorganisms in anaerobic digestion (AD) [7].

An appropriate pre-treatment method is needed to disrupt the ordered structure of lignocellulosic materials. Several pre-treatment methods have been investigated in order to increase the biomethane (methane fraction of biogas) yield of lignocellulosic materials. These methods include thermal, chemical, biological, and enzymatic pre-treatment methods [8]. Biological pre-treatment typically involves incubating the substrate aerobically with fungi that will partly delignify it. This is a low-temperature process with minimal production of any inhibitors. The main barrier for commercial applications is that a relative long treatment time is needed [9]. During enzymatic pre-treatment, commercial enzymes that degrade polysaccharides and/or lignin are applied. However, the relatively high cost of enzymes restricts industrial applications [10]. Other pretreatments involve application of high temperatures and/or chemicals. Examples are alkali [11] or acid pre-treatment [12], wet explosion and steam pre-treatment [13]. Steam explosion is a method where high temperature steam is used to penetrate the lignocellulosic structure. After a specific residence time, the substrate is released into atmospheric pressure, resulting in an expansion of the steam which rip the fibers apart. Steam explosion is known as an energy/cost-effective method to pretreat lignocellulosic materials to reduce cellulose’s crystallinity and increase the surface area for enzymatic activities of different microorganisms in AD [14–17]. However, steam explosion may also have some negative impact on biomethane production. At high temperatures, parts of the C_5_ and C_6_ sugars in the biomass may be converted to furfural and hydroxymethylfurfural (HMF), respectively, which may inhibit the AD process [18, 19]. A clear advantage of stem explosion is that no chemicals need to be added and that the process has reached industrial scale, then often termed a thermal hydrolysis process (THP) [20].

Lignocellulosic materials have a low content of nutrients (such as nitrogen), and this type of biomass cannot alone sustain microbial growth in a continuous flow reactor [21, 22]. Co-digestion of lignocellulosic materials with nutrient-rich substrates (e.g., manure) not only dilutes the possible inhibitory effect of pre-treated lignocellulosic substrates but also balances the carbon and nitrogen ratio. In this way, the organic loading rate (OLR) and methane yield of a digester can be increased [22].

AD is a complex four-step anaerobic biological process including hydrolysis, acidogenesis, acetogenesis and methanogenesis where the methane is the final electron sink in absence of inorganic oxidant such as nitrate, ferric iron, or sulfate [23, 24]. In the last step, methane is produced from a few intermediate substrates as follows:

1 $$\begin{aligned} & {\text{CH}}_{3} {\text{COO}}^{ - } + {\text{H}}_{{2}} {\text{O }} \to {\text{CH}}_{4} + {\text{HCO}}_{3}^{ - } \\ & \Delta G \, = \, - 31{\text{ kJ/mol}} \end{aligned}$$

2 $${\text{CH}}_{3} {\text{COO}}^{ - } + {\text{4H}}_{{2}} {\text{O }} \to 2{\text{HCO}}_{3}^{ - } + {\text{4H}}_{{2}} + {\text{H}}^{ + } \quad \Delta G \, = \, + 104{\text{ kJ/mol}}$$

3 $${\text{4H}}_{{2}} + {\text{HCO}}_{{3}}^{ - } + {\text{H}}^{ + } \to {\text{CH}}_{{4}} + \, 3{\text{H}}_{{2}} {\text{O}}\quad \Delta G \, = \, - 135{\text{ kJ/mol}}$$

The energy efficiency of AD depends on several factors, including, but not limited to, the hydrolysis process and a close partnership between syntrophic bacteria and methane-producing archaea. Depending on the nature of the substrate, one of the main AD steps can act as the rate-limiting step [25]. Physicochemical parameters such as CH_4_ content in biogas, pH level, volatile fatty acid (VFA), and ammonia concentration may provide information regarding the overall performance and stability of the AD; however, physicochemical parameters do not directly inform about the microbial community structure and possible changes in it.

During normal operational conditions, acetate degradation (Eq. 1) is typically the main pathway for biomethane production, and acetate consumers (e.g., *Methanosaeta* and *Methanosarcina*) typically contribute to over 70% of the total methane production. However, in stress conditions such as high ammonia concentration (reported from 1.4 to 14 g/L) [26] or high temperature, an acetate oxidation reaction (Eqs. 2 and 3), known as syntrophic acetate oxidation, followed by hydrogen consumption via hydrogenotrophic archaea (e.g., *Methanomicrobiales*, *Methanobacteriales*, *Methanococcales*, *Methanoculleus* and some family members of *Methanosarcina*) can take over the biomethane production process (the overall Gibbs energy of the acetate oxidation route is Δ*G* = − 31 kJ/mol) [24, 26]. A close partnership between acetate oxidizing bacteria and hydrogenotrophic archaea is needed to produce methane from the syntrophic acetate oxidation pathway (Eqs. 2 and 3) [26]. In addition to these two pathways, electrons can be transferred from bacteria to archaea through direct interspecies electron transfer (DIET). In DIET (9H^+^ + 8e^−^ + HCO_3_^−^ → CH_4_ + 3H_2_O), instead of hydrogen (or formate), electrons move through biological wires (e-pili) or conductive materials making it thermodynamically more favorable [27].

The stability of the microbial community is a critical factor in the methane production process and requires high adaptability of the microbial community during change in process conditions, especially for methanogens, due to a lack of functional redundancy [28]. At the same time, the diversity of various bacteria can provide stable hydrolysis, acidogenesis, and acetogenesis. In addition to advanced molecular biological techniques, stable ^13^C isotope analysis of the biogas can rapidly reflect stability/disruption of the microbial community, especially methanogens, in laboratory- or full-scale biogas digesters [29]. This is known that the ratio of ^13^C and ^12^C in the produced biogas may change depending on whether methane has been produced through the acetoclastic or hydrogenotrophic pathway. This is for example indicated by change in $\delta^{13} {\text{C}}_{{{\text{CH}}_{{4}} }}$ produced by CO_2_ reduction where the $\delta^{13} {\text{C}}_{{{\text{CH}}_{{4}} }}$ is lower comparing to the $\delta^{13} {\text{C}}_{{{\text{CH}}_{{4}} }}$ produced from acetate [30, 31]. Thus, carbon isotope ratio in the biogas can be linked to the methane-producing pathway and the microbial community composition [32].

Laukenmann et al. [31] developed a correlation between microbial community and $\delta^{13} {\text{C}}_{{{\text{CH}}_{{4}} }}$ that could predict the dominant methane-producing pathway [31]. Previous studies have linked the abundance of methanogens and apparent fraction factors [33, 34]. Generally, an *α*_c_ > 1.065 represents a microbial community with very high abundance of hydrogenotrophic methanogens (CO_2_ oxidation with H_2_ is the main pathway for methane production), while an *α*_c_ < 1.025 indicates an acetoclastic-dominant pathway for methane production [35].Culture adaptation can improve biogas production due to achieving a more beneficial microbial community composition and a more robust process [9]. Adaptation of the microbial community involves a gradual change of an operational parameter, such as type of feedstock, organic loading rate, temperature, and other factors [4]. A shift in the nutrient composition added to a continuous flow reactor will in most cases result a shift in the microbial community.

Since the working volume of a reactor is always constant, some microorganisms will leave the reactor from the outlet due to the feeding. Growth and reproduction among microorganisms constantly happen but at different speeds. The change of substrate loading, or the introduction of a new substrate may slow down or speed up the development and replication of various organisms, resulting in the washout or domination of different species. Theoretically, 1 HRT exchanges the total volume of a reactor, and it is typically recommended that 3 HRTs should be applied to give the microbial community sufficient time to adapt to the new conditions and reach a new equilibrium.

To the authors’ best knowledge, culture adaptation for improved biomethane production from steam-exploded birch wood (SEBW) and cow manure (CM) has not been investigated previously. This would potentially involve improved tolerance of the microbial community against inhibitory compounds in SEBW and optimization of the methane production pathway. To investigate this, changes in the microbial community during the adaptation process were in this study monitored by 16S rRNA gene sequencing. Moreover, the adapted culture was tested for possible improvement in the tolerance against potential inhibitors produced during the steam explosion (e.g., furfural and HMF). Possible changes in the main methane production pathways were monitored using the ^12^C/^13^C isotope ratio of the produced biogas. Finally, correlations between changes in the methane production pathway and the microbial community composition were investigated.

## Materials and methods

### Raw material

#### Birch wood

The birchwood (*Betula pubescens*) was harvested in Trøndelag county in the west part of Norway. The birchwood was grounded to pieces (Al-Ko Compost grinder Easy Crush MH 2810) to pieces of 15 to 30 mm. The wood chips were kept at room temperature for 2 weeks to reduce the moisture content (see Table 1 for composition data).

**Table 1:** Physical and chemical characterization of the birch wood, steam-exploded birch wood, cow manure, and start-up inoculums

Sample	Size mm	TS%	VS%	Lignin^a^ %^a^	Glucose^b^	Xylan^b^	Arabinan^b^	Galactan^b^
Birch wood	15–30	81	70.9	20	36.4	16.5	1.1	1.5
Cow manure		16.6	12.5	26.8	27.2	16	5.7	–
Steam exploded birch wood	< 3	43	37.2	40.3	42	14.8	0.8	1.2
Inoculum 1	–	4.7	3.67	–	–	–	–	–
Inoculum 2	–	6.7	5.4	–	–	–	–	–

See “Analysis” for details about the used analytical methods

^a^The lignin content presented based on the dry matter content

^b^Carbohydrates are presented as mass fraction in % (g/g Dry matter) × 100

#### Cow manure

Cow manure (CM) was collected from a livestock farm belonging to the Norwegian University of Life Science at Ås, Norway. The CM was diluted with tap water to a final total solid content of 6% and volatile solid content of 4.4%.

### Inoculum

The microbial culture was a mixture of two different inoculums with a ratio of 4:2. The first inoculum was obtained from a full-scale continuous flow mesophilic (40 °C) biogas plant (Biokraft, Trøndelag, Norway). The primary substrate for this reactor was fat- and protein-rich substrates (fish silage). The total solid (TS) content of the inoculum was 4.7%, and the volatile solids (VS) content of the inoculum was 3.67%, with a pH of 7.8.

The second inoculum (pH: 7.6) was collected from a lab-scale CSTR. CM was the feedstock for this reactor which was also operated at mesophilic conditions (38 °C). The TS and VS of the inoculum were 6.7% and 5.3%, respectively. Prior to the experiment, the inoculums were incubated anaerobically at 40 °C for 10 days to reduce endogenous biogas production.

### Steam explosion pre-treatment

Steam explosion (SE) pre-treatment was performed as described previously [15, 36] using an SE rig designed by Cambi AS (Asker, Norway) and located at the Norwegian University of Life Science in Ås, Norway. Briefly, 800 g of woodchips were loaded into the preheated 20 L vessel. Steam was quickly added to reach a temperature of 220 °C in the vessel and the woodchips were kept at this temperature for 10 min. Then, the outlet valve was opened, quickly pushing the pre-treated biomass out of the vessel into an expansion tank and reducing pressure to atmospheric leading to a disruption in the woodchip’s structure. The steam-exploded birch wood (SEBW) was packed in vacuum bags and stored at − 20 °C before further use. Table 1 shows the physical and chemical characteristics of inoculum and substrates used in this study.

### Digester operation

Two CSTR bioreactors (Dolly, Belach Bioteknik AS, Sweden) with 6 L working volume were operated at 40 °C for 120 days. The digesters were stirred using axial impellers at 80–100 rpm. The start-up inoculum was a blend of the first and second inocula, described in “Microbial community analysis” (mixing ratio was 4 L:2 L, respectively). The reactors were fed with liquid cow manure for the first five days to increase the alkalinity of the CSTR reactors and protect the process from being soured by VFAs. Afterward, the digesters were fed every day with mixture of CM and SEBW (Table 2), keeping the operation volume constant in the reactor by removing liquid from the reactor equal to the inlet volume. These two reactors operated in parallel to adapt the microbial community to the carbon-rich SEBW during the co-digestion with CM. After reaching a stable condition, in one of the reactors (R_0_) the organic loading rate (OLR) was kept constant at 1.8 g VS/L-day for over 80 days to observe and compare the microbial community dynamics during the adaptation period. The OLR in the other reactor (R_1_) was ramped up from 1.8 to 3.2 g VS/L-day to investigate the tolerance of the microbial community against the overloading. The operational condition and OLR of each reactor during the adaptation period are provided in Table 2.

**Table 2:** Operational condition of the CSTR reactors

	R_0_						R_1_					
	Day 0	Day 1–5	Day 6–35	Day 36–65	Day 66–95	Day 95–120	Day 0	Day 1–5	Day 6–35	Day 36–65	Day 66–95	Day 95–120
SEBW (g/Day)	0	0	7.7	16.3	16.3	16.3	0	0	7.7	16.3	28.6	37.2
CM (mL/day)	0	72	100	150	150	150	0	2.4	100	150	200	200
OLR (g VS/L∙day)	0	0.4	1	1.8	1.8	1.8	0	0.4	1	1.8	2.7	3.2
C:N ratio	–	15.57	26.34	30.71	30.71	30.71	–	15.57	26.34	30.71	35.33	40.95
pH	7.6	8.2	7.6	7.38	7.47	7.42	7.72	8.24	7.61	7.4	7.33	7.01
NH_4_ (g/L)	1.88	2.87	1.65	0.67	0.59	0.56	1.7	2.97	1.44	0.77	0.58	0.52
Acetic acid (mg/L)	186	281	524	451	407	166	296	315	267	614	714	841
Propionic acid (mg/L)	110	136	166	214	335	121	158	121	90	301	384	731
Iso-butyric (mg/L)	n. d	58	104	37	173	65	59	69	87	93	311	489
Butyric (mg/L)	n. d	41	85	104	398	236	n. d.	13	21	580	417	n. d.
Iso-valeric (mg/L)	8	14	14	2	4	0	5	6	18	40	52	n. d.
Valeric (mg/L)	n. d.	n. d.	n. d.	n. d.	2	n. d.	n. d.	n. d.	n. d.	84	n. d.	n. d.
Total VFA (mg/L)	304	531	892	808	1318	422	517	524	485	1712	1878	2061

The values have been provided as the average value measured over the feeding period

See “Analysis” for details about the used analytical methods

### Biomethane potential

#### Maximum biogas production rate

The improvement of biogas production rate during the adaptation period was investigated through a method previously described by Østgaard et al. [37]. Briefly, 40 mL (1.42–1.46 g VS) of effluent samples after each HRT (day 6, 35, 65, 95 and 120) was directly transferred to a 100 mL medical syringe with an on/off valve. 1 mL CM mixed with 1 g SEBW (0.36–0.37 g VS) was added to the syringes. Headspace air was removed to ensure anaerobic condition. The syringes were incubated for 15 days at 40 °C, and each experiment was conducted in triplicate and three control syringes without any added substrate were provided for each sampling point. The biogas production in the syringes was measured using the volume gauge on the syringe. Daily sample from the syringes was taken to analyze the gas composition by a gas chromatograph (GC) (SRI 8610C, SRI Instruments, USA). The biogas produced in the corresponding control syringes were subtracted to determine the methane production from substrate conversion in each sampling point.

#### Hydroxymethylfurfural and furfural mixture preparation and BMP test

Initial (day 6) and final (day 120) inocula from R_0_ were employed to investigate the effect of culture adaptation on overload of inhibitory elements. Briefly, a 50% (wt %) mixture of HMF and Furfural was used (i.e., 5 to 50 mM furfural and 3.9 to 40 mM HMF). Four different concentrations (i.e., 1, 2, 3, 4, and 10 g/L) of the mixture were combined with 1 g VS of SEBW–CM mixture in batch reactors. The mixtures were digested anaerobically in medical flasks with a total volume of 500 mL and closed with a rubber stopper and aluminum screw caps. Then, the headspace (300 mL) was flushed with pure nitrogen to ensure an anaerobic environment. AD of substrates was conducted in triplicate and included the control (only SEBW–CM). The AD was performed at 40 °C for 30 days in an Infors Minitron shaking incubator (Infors, Bottmingen, Switzerland).

### Analysis of microbial community structure

#### DNA extraction and quantification

Liquid samples were periodically (after each HRT) collected from the reactors and stored in freezer. Microbial community analysis was carried out by DNASense ApS (Aalborg Øst, Denmark). For DNA isolation, the FastDNA spin kit for soil (MP Biomedicals, USA) was employed. In short, 500 μL of sample was added to a lysing matrix E tube including 480 μL sodium phosphate buffer and 120 μL MT buffer. Bead beating to crush the cells was conducted at 6 m/s for 4 × 40 s [38]. The ultimate DNA purification was determined by Gel electrophoresis using Tapestation 2200 and D1000/High sensitivity D1000 screentapes (Agilent, USA). The DNA concentration was determined using Qubit dsDNA HS/BR Assay kit (Thermo Fisher Scientific, USA).

#### 16s RNA gene amplicon sequencing

Amplificon libraries for bacteria/archaea 16S RNA gene (i.e., region 4 abV4-C) were prepared based on illumine protocol [39]. 10 ng of extracted DNA was used as template for the PCR amplification of bacteria/archaea 16S RNA variable gene region 4 (abV4-C) with primers of 515FB (GTGYCAGCMGCCGCGGTAA) and 806RB (GGACTACNVGGGTWTCTAAT) [40] designed according to illumine [39]. After extracting the sequencing libraries from PCR, the results were quantified and purified. The purified amplicons were pooled in equimolar concentration and diluted to 2 nM and then paired-end sequenced (2 × 300 bp) on a illumine MiSeq platform (Illumina, San Diego, USA) following the standard guidelines for preparation and loading samples on MiSeq. In other to overcome the complexity issue often observed with amplificon samples, > 10% PhiX control library was spiked.

### Measurement of the stable isotope composition of the biogas

Biogas samples for carbon isotope analyses were collected in a 1 mL gas-tight syringe before feeding the reactors (5 parallel samples from reactors) at each sampling point for the 16S rRNA analyses. Through mixing zero-air (20.9 ± 0.2% O_2_ in N_2_, Air liquid AS, Norway) with 0.2 mL of the collected gas, the methane (CH_4_) and carbon dioxide (CO_2_) concentrations were maintained below 1000 ppm. A Picarro G-2201 CH_4_/CO_2_ isotope analyzer (CA, USA) was employed to analyze the ^13^C in samples. Along with the samples, different ALPHAGAS™ (Airgas, Ca, USA) standard gases with known ^13^C isotopes were used to adjust the calibration curves in the isotope analyzer. For this purpose, 20 mL of gas sample was automatically injected into the cavity ring-down spectroscopy (CRDS) to detect the trace elements at 45 °C. Afterward, the measured isotopes were adjusted by the calibration gas to determine the ^13^C and ^12^C of the samples.

δ^13^C indicates the ratio between the rare and abundant carbon isotopes and usually is expressed as follows:

$$\delta ^{{13}} {\text{C }} = {\text{ }}\left\{ {\left[ {{{\left( {{}^{{13}}{\text{C}}/{}^{{12}}{\text{C}}} \right)_{{{\text{sample}}}} } \mathord{\left/ {\vphantom {{\left( {{}^{{13}}{\text{C}}/{}^{{12}}{\text{C}}} \right)_{{{\text{sample}}}} } {\left( {{}^{{13}}{\text{C/}}{}^{{12}}{\text{C}}} \right)_{{{\text{standard}}}} }}} \right. \kern-\nulldelimiterspace} {\left( {{}^{{13}}{\text{C/}}{}^{{12}}{\text{C}}} \right)_{{{\text{standard}}}} }}} \right] - 1} \right\} \times 1000\,{ \permil}$$

where *δ* in the formula is expressed as part per million (‰). To assess variation of methane production pathway during AD of SEBW and CM, the apparent carbon isotope fraction (αc) was calculated as proposed by Whiticar et al. [34]:

$$\alpha_{{\text{C}}} = \, \left[ {\left( {\delta^{13} {\text{CO}}_{{2}} \, + {1}000} \right) \, / \, \left( {\delta^{13} {\text{CH}}_{4} + {1}000} \right)} \right]$$

### Analysis

Volume of daily biogas production calculated using a water displacement gas meter with 38 mL volume over 24 h. The methane and carbon dioxide content of biogas (vol %) measured every day by GA2000 Landfill Gas Analyzer (Geotechnical Instruments Ltd., UK). The concentration of TS, VS, and pH level were determined according to International and European Organization for Standardization (ISO 10390: 2005; ISO 11465: 1993; NS-EN 15935: 2012.”). For ammonium (NH_4_) concentration analysis, the samples were collected weekly and diluted 10 times. The NH_4_ concentration was measured using photometric 5.2–103 mg/L cell test (Merck group, Germany) and Spectroquant^®^ Prove 100 spectrophotometer. The VFAs of the samples were analyzed by a Rezex RFQ high-performance liquid chromatograph (HPLC) (Phenomenex, Torrance, Ca, USA) equipped with 3000RC column and operated at 85 °C and UV detection at 210 nm (Dionex, Sunnybale, CA, USA). Prior to analyses of VFAs, the samples were diluted with sulphuric acid. For quantification, VFA standards were applied. Sugar composition of BW and SEBW was determined based on NREL/TP-510-42618 [41]. In brief, acid hydrolysis was used to generate soluble sugars and acid-insoluble lignin residues, where the latter was dried overnight at 105 °C in and weighed to obtain the acid-insoluble lignin (Klason lignin) content. The soluble sugars were analyzed by high-performance anion-exchange chromatography with pulsed amperometric detection (HPAEC-PAD) (Dionex ICS-6000, Dionex, Sunnyvale, CA, USA). Separation of soluble sugars was achieved utilizing a 2 × 150 mm Dionex CarboPac PA-210-Fast-4 µm column (Thermo Scientific) connected to a 2 × 30 mm guard column of the same type. The operational flow was 200 µL/min and the sample loop volume was 0.4 µL. The columns were kept at 30 °C.

## Results and discussion

### Improved biomethane production

#### CSTR experiments

**Fig. 1:**
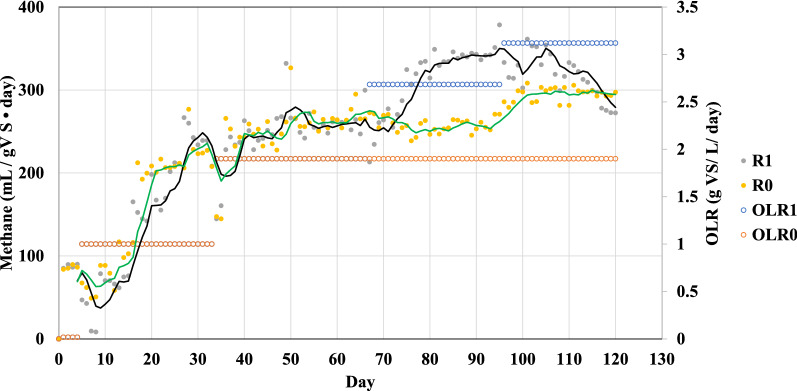
Daily methane production and organic loading rate for R0 and R1. Lines indicate methane production as a 2-day moving average (R0 in green and R1 in black)

Two anaerobic digesters were employed to investigate the potential improvement of biomethane production from SEBW during co-digestion with CM. The steam explosion disrupted the birch wood's structure, converting it to a dark brown solid with few visible fibers. The results of biomethane production from the CSTR reactors, operated for 120 days, are provided in Fig. 1.

Start-up inocula were fed a small amount of liquid CM (0. 4 g VS) for five days, as indicated in Table 2. The liquid CM was employed to activate the microorganisms and increase the system’s alkalinity. This way, the reactors were protected from possible soreness due to VFA release from the degradation of lignocellulose in the first week. After 5 days, the OLR of the reactors was increased to 1 (g VS/L/day). During the experiment, the OLR of R_0_ was increased only one level from 1 to 1.82 (g VS/L/day), while the OLR of R_1_ was ramped in three levels from 1 to 3.12 (g VS/L/day). In the first week, the daily methane production was reduced from over 80 to 50 (mL/g VS/day). Minimum daily methane production was recorded on day 6 due to high ammonium content in both reactors resulting in elevated pH of over 7.9. The inhibiting rate for ammonia concentration varies between 1.4 and 14 g/L, which can cause a maximum of 50% reduction in methane production [26]. By changing feed to a carbon-rich substrate (SEBW) and rising OLR to 1.8 (g VS/L/day) on day 35, the concentration of VFAs increased in the systems (over 0.8 g VFA/L) and balanced the pH [42] while methane production extended to over 250 (mL/g VS/day).

The OLR in R_0_ was kept constant from day 35. Consequently, the methane production in R_0_ was relatively stable at around 250 ± 17 (mL/g VS/day) until day 95, when the methane production in R_0_ started to increase and reached over 300 (mL/g VS/day). Improved daily methane production with constant OLR in R_0_ (day 95 compared to day 25) may suggest that a long-term adaptation affects the microbial community through specializing them in converting available substrates (e.g., different types of carbohydrates) to methane in a shorter time. In addition, there might be some changes in electron transfer processes in the systems making it more stable for methane production [43].

In contrast, the OLR of R_1_ was increased twice, on days 65 and 95. As a result of an increase in OLR, the biomethane production in R_1_ reached a maximum amount of over 365 (mL/ g VS/ day) when the OLR was 2.7 g VS/L/day).

By increasing the OLR to 3.28 g VS/L/day, the biomethane production in R_1_ was reduced, and after day 105 had a downward trend. The lower biomethane production rate was associated with a drop in pH due to VFA accumulation in the same period (See Table 2), reflecting disturbances in the metabolic pathways of the microbial community. The maximum daily methane production rate from SEBW and CM (365 mL/ g VS day) was 70% compared to the maximum theoretical methane production rate of SEBW–CM mixture (522.3 mL/g VS) when the OLR in R_1_ was 2.7 (g VS/L/day) [44]. This biomethane production rate in the CSTR reactor was much higher than methane production (230–240 mL/g VS) reported from anaerobic co-digestion of cow manure and steam-exploded hardwood in continuous flow CSTR reactor [45, 46]. The daily biomethane production rate from SEBW and CM was even higher than previously reported biomethane produced from enzymatic pre-treated SEBW with enzyme cocktails in batch experiments [15]. This suggests that the 2.7 (g VS/day) can be a suitable OLR (Table 2). In the final step of the experiment, lower methane production rate was accompanied by accumulation of VFAs (propionate) [42].

#### Batch AD

**Fig. 2:**
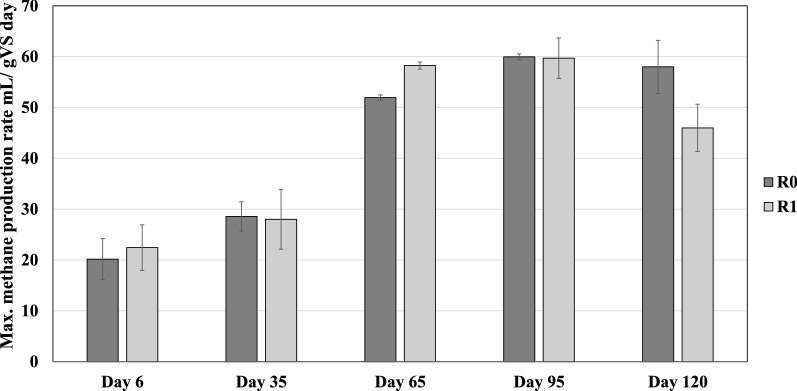
Maximum methane production rates from SEBW–CM in batch tests using digestates collected from reactors R0 and R1 at different times as inocula

The maximum biomethane production rate of different inocula was investigated using 40 mL of effluent inoculums at the end of each feeding period (days 6, 35, 65, 95, and 120). The batch reactors (in triplicate) were fed with 0.36–0.37 g VS of mixed SEBW and CM. The mixture was digested for 15 days at 40 °C to compare the kinetic biogas capacity of the digester microbial population. As shown in the Fig. 2, the maximum biomethane production rate of experiments with sludges from R_0_ significantly increased from 20.17 ± 4 (mL/g VS/day) on day 6 to 60 ± 0.6 by day 95 and remained approximately unchanged until the end of the experiment. The maximum biomethane production rate of the samples with sludges from R_1_ increased from 22.4 ± 4 on day 6 to 59.7 ± 4 (mL/g VS/day) at day 95. However, the maximum biomethane production rate in batch test using R_1_ effluent on day 120 was reduced to 46 ± 4.6 (mL/g VS/day) (Fig. 2). The results indicate that after a long-term adaptation the microbial community have changed to increase degradation kinetics in different sampling points, where maximum biomethane production rate of the inoculums from final days (e.g., 95 and 120) were significantly higher than that in initial inoculum (Fig. 2).

#### Adaptation effects on microbial community resistance against inhibitors

Steam explosion of lignocellulosic materials, including birch wood, can increase the concentration of Furfural and HMF that act as inhibitors in AD [16, 19]. An increase in OLR of SEBW increases the concentration of inhibitors and may reduce the biomethane production yield. It has been reported that methanogens such as *Methanococcus deltae*, *Methanococcus thermolitotrophicus*, *Methanobacterium thermoautotrophicum*, *Methanosarcina barkeri* and *Methanococcus ruminantum* will not grow in the presence of 5-methyl- and 2-methylfurfurals. However, it is observed bacteria such as *Methanococcus* sp. strain B capable of growth on HMF and 2-methylfurfural as sole carbon source [47]. In a later study by Belat et al., it was reported that *M. deltae* grow in the presence of various concentrations of furfural [48].

**Fig. 3:**
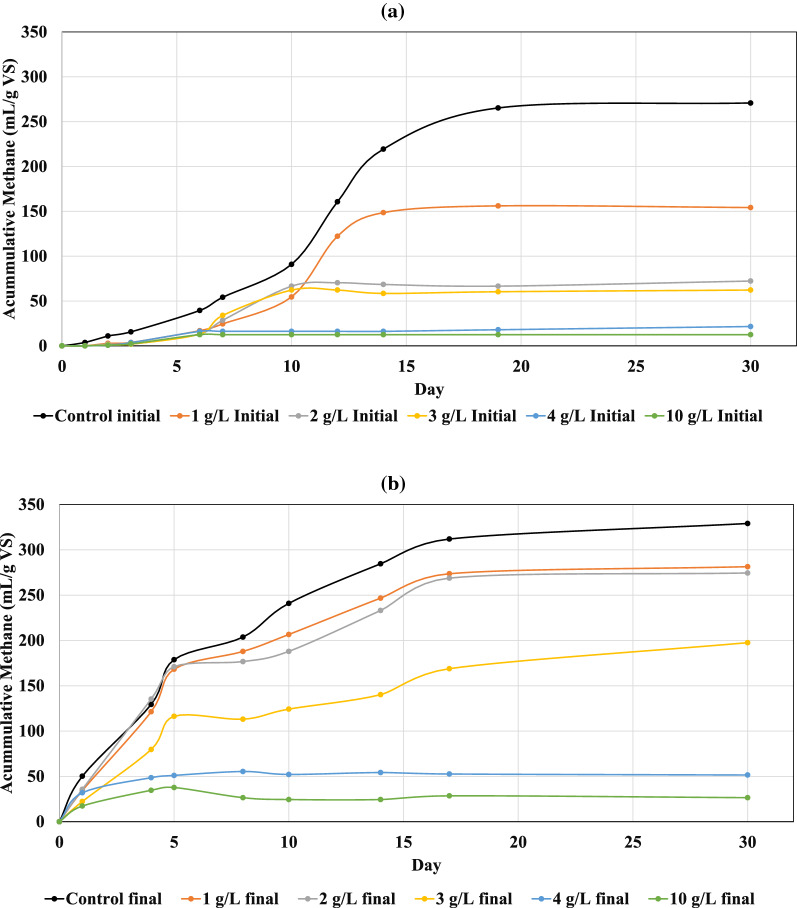
Methane production from SEBW–CM in batch tests using day 6 (**a**) and day 120 digestates (**b**) from R0 as inocula in the presence of different HMF–furfural concentrations (1–10 g/L)

Initial (day 0) and final inoculum (day 120) from R_0_ were employed in the BMP test to assess the resistance of the microbial community against an increase in the concentration of inhibitors. SEBW–CM mixture (1 g VS) combined with different HMF–furfural mixture concentrations and digested for 30 days at 40 °C as described in “Hydroxymethylfurfural and furfural mixture preparation and BMP test”. The biogas production yield of various experiments using initial and final inoculum is presented in Fig. 3. The accumulated methane production from controls (i.e., samples with only SEBW–CM mixture) after 10 days using the final inoculum reached 240 (mL/g VS), which is almost 2.6-fold higher than that in similar experiments using initial inoculums (90 mL/g VS). The accumulative methane from the control samples after 30 days was 270 ± 10 and 329 mL/g VS for initial and final inoculum, respectively. Experiments with the final inoculum, including 1 and 2 g/L HMF–furfural mixture, yielded higher biomethane than the initial inoculum’s control experiment (i.e., 281 and 274 mL/g VS, respectively). Adding 1, 2, and 3 g/L of HMF–furfural mixture to experiment with the initial inoculum significantly reduced the methane production yield to 154, 72, and 65 mL/g VS., increasing the concentration of the inhibitor to 4 and 10 g/L caused a significant reduction in all experiments using either of inoculums. The results show that adding inhibitors beyond the microbial culture tolerance (over 2 g/L) has inhibited the methane production in all the samples; however, this effect for experiments with final inoculum is not significant, especially when the concentration of the inhibitors is up to 2 g/L. This may suggest that the microbial culture adapted to not only digestion of the feedstock but also the presence of a higher concentration of inhibiting elements [48–50].

Several studies have reported that HMF has a negative effect on fermentative H2 production [51–53]. However, low concentrations of HMF have been reported to contribute to hydrogen production in anaerobic fermentation [54]. Studies have shown that when HMF concentration was 0.60 g/L, no inhibition of H2 formation was observed and biogas production was maximal. When the HMF concentration in the experimental reactors exceeded 0.60 g/L, the H2 production was gradually inhibited. At HMF concentrations above 0.9 g/L, not only the production of hydrogen was inhibited, but it also impacted the biofilm structure and the microbial community dynamics [53]. HMF concentrations over 2 g HMF/L can completely stop the methane production [55–57]. HMF concentrations over 2 g HMF/L can completely stop the methane production [55–57]. It has been claimed that the furfural concentration below 1 g/L does not have a significant effect on biogas production rate [55]. This may suggest that the methane production rate in a lower concentration of furfural and HMF mixture is most probably inhibited due to the presence of HMF rather than the furfural; however, this hypothesis should be further investigated individually.

### Microbial community analysis

Microbial sequence analysis showed a change in the microbial community during the experimental period. The relative abundance of different bacteria and archaea is provided in Fig. 5. Over 90% of bacterial composition in start-up inoculum consisted of *Firmicutes* (73.6%), *Bacteroidota* (5,9%), *Cloacimonadota* (5%), *Caldatribacteriota* (4.2%), and *Proteobacteria* (3,7%), which is in agreement with other studies, reporting that in nearly all methane-producing microbial reactor populations, species from Firmicutes and Bacteroidetes are dominant [58–60]. On day 6, bacterial community composition in R_0_ and R_1_ slightly changed to *Firmicutes* (82.3 and 81%), *Bacteroidota* (6.3 and 8.1%), *Caldatribacteriota* (4.3 and 3.6%), and *Acidobacteriota* (2.1, 1.9%), respectively. On day 120, when the culture was adapted to SEBW and CM, the bacterial community composition in R_0_ and R_1_ had significantly changed compared to the start-up inoculum. In R_0_ and R_1_, the relative abundance of *Firmicutes* (42.2, 41.2%) species was notably reduced compared to the initial inoculum. At the same time, the relative abundance of different species in R_0_, including *Bacteroidota* (22%), *Cloacimonadota* (10.7%), *Fibrobacterota* (6.5%), *Acidobacteriota* (5.5%), *Caldatribacteriota* (3%) and *Synergistota* (3%) gradually increased during the adaption period reaching to its maximum on day 120. Reactor R_1_ experienced similar changes during the adaption period when relative abundance of *Cloacimonadota* (15.9%), *Bacteroidota* (15.2%), *Fibrobacterota* (6.3%), *Acidobacteriota* (5.5%), *Caldatribacteriota* (4.4%) and *Synergistota* (3.7%) were changed as a function OLR.

**Fig. 4:**
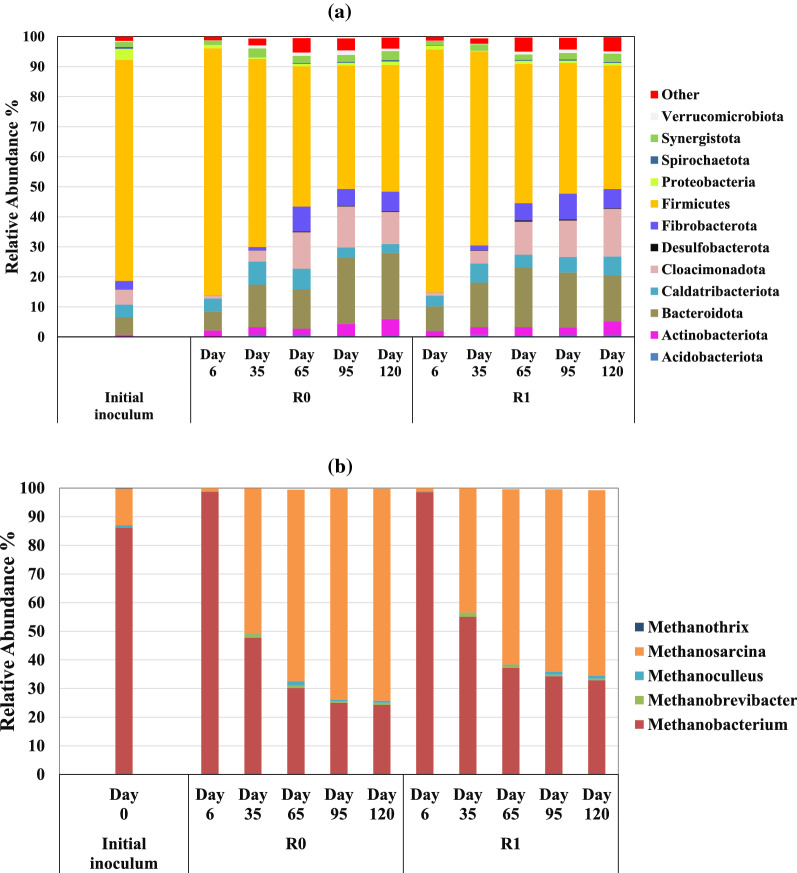
Relative abundance of bacterial phyla (**a**) and archaeal family (**b**)

*Methanobacterium* (86.2%), *Methanosarcina* (12.2%), *Methanoculleus* (0.8%) and *Methanothrix* (0.2%) were the most abundant methanogenic archaea in the start-up inoculum, highly dominated by hydrogenotrophic methanogens. This is in accordance with the parameters in the biogas plant where the inoculum was sourced, which were operated at high nitrogen levels typically related to hydrogenotrophic methanogenesis and syntrophic acetate oxidation (SAO). Especially *Methanoculleus* is known to work in syntropy with SAO bacteria to generate methane. *Methanothrix*, however, is mainly an acetoclastic genus, and the presence of this methanogenic group illuminates the fact that in most reactors’ methane is formed from both hydrogen and acetate, although dominated by one of the reaction pathways. Initially, on day 6, the relative abundance of *Methanobacterium* increased to 98.7 and 98.5% in R_0_ and R_1_, respectively, while the other methanogens had minimum abundance in the culture. From day 6 on, *Methanobacterium* abundance was reduced, and on day 120 this genus reached a minimum of 24.3 and 32.9% in R_0_ and R_1_, respectively. In contrast, the relative abundance of *Methanosarcina* and *Methanoculleus* increased to 74.1 and 0.7% in R_0_. These values for R_1_ were 64.7 and 1.1%, which is significantly higher than that on samples from day 6 (0.1 and 0.2%, for *Methanosarcina* and *Methanoculleus*, respectively). The members of the genus Methanosarcina are composed of both acetoclastic and hydrogenotrophic methanogens. In general, the results indicate that the dominating reaction pathway for methane production changed from hydrogenotrophic to acetoclastic as the operational parameters including substrate chemical properties was changed (Fig. 4).

**Fig. 5:**
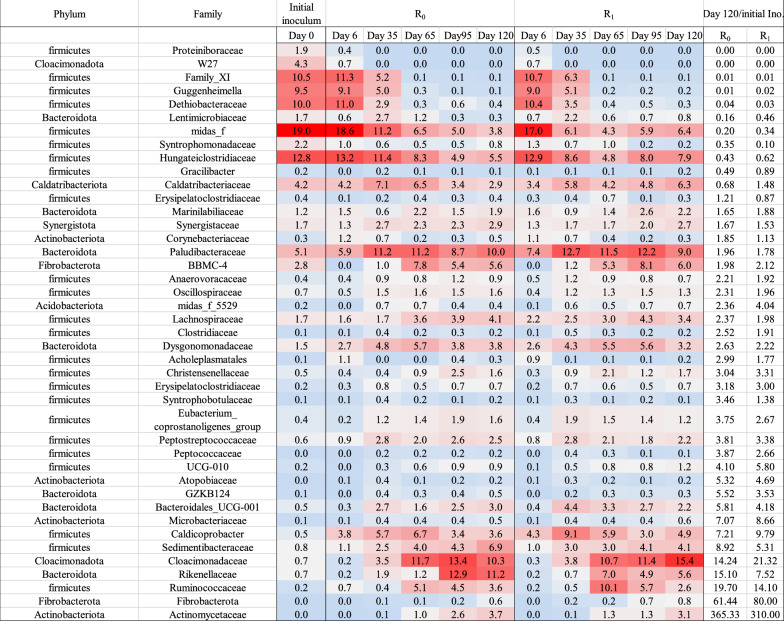
Heat map of the most abundant bacterial families. Colors indicate low (light blue), medium (light gray), and high (red) abundance. The two columns to the right show abundance ratio between day 120 and initial sample. The table is sorted from low to high abundance ratios

Figure 5 presents a heatmap of the 42 most abundant bacterial families sorted from most significant relative abundance reduction (on top) to largest relative abundance increases on day 120. The predominant species among all the bacterial communities was *Firmicutes*. During the adaptation period, the relative abundance of several family members belonging to *Firmicutes*, *Bacteroidota*, *Cloacimonadota*, *Fibrobacterota*, *Acidobacteriota*, *Caldatribacteriota*, and *Synergistota* changed. From that, the relative abundance of W27 belonging to *Cloacimonadota* phylum, *Clostridia_Family_XI*, *Guggenheimella*, *Dethiobacteraceae*, *Syntrophomonadaceae*, uncultured *Firmicutes* species (from *Limnochordia* and D8A-2 class) and *Hungateiclostridiaceae* was reduced by 100, 99, 98, 96, 65–90, 60–80 and 30–50%, respectively, as stated in Fig. 5.

Even though methane production from acetate is thermodynamically more favorable compared to hydrogenotrophic methanogenesis pathway, under high ammonia conditions the acetate oxidation pathway can be the main methane production pathway [26]. The start-up inoculum contained fat- and protein-degrading microorganism. Methane produced during the first week, when the pH was high and *Methanobacterium* was the dominant archaea, might be linked to an active syntrophic acetate oxidation pathway. Along with hydrogenotrophic methanogens, the relative abundance of Firmicutes in both reactors was over 80%. Over 90% of *Firmicutes* constituted of most abundant families were members of uncultured bacteria from *Limnochordia* and D8A-2 class, *Hungateiclostridiaceae*, family members of *Clostridia* class (*Family_XI*), *Dethiobacteraceae*, *Guggenheimella*, *Syntrophomonadaceae*, and *Caldicoprobacter*, [61–63] most of them related to SAO.

In a recent study, a genetic analysis of 20 biogas plants indicated that the family members of *Limnochordia* can be a potential syntrophic partner of acetate/hydrogen consumers at elevated nitrogen concentrations [64, 65]. Members of *Hungateiclostridiaceae* (e.g., *Ruminiclostridium*) are hydrolytic bacteria producing extracellular cellulosomes to depolymerize cellulose and polysaccharides [66]. Relative abundance of *Hungateiclostridiaceae* was reduced during the adaptation period as a result of a reduction in relevant abundance of Firmicutes, yet *Hungateiclostridiaceae* family members were the most abundant families within the *Firmicutes* phylum on day 120 [67]. This suggests that the *Hungateiclostridiaceae* play an essential role in hydrolysis of lignocellulosic feedstocks. Members of *Clostridiales* Family XI are present in anaerobic digesters accomplishing diverse metabolic functions including hydrothermal hydrolysis of amino acids at high ammonia conditions [68, 69]. *Syntrophomonadaceae* and *Dethiobacteraceae* are previously reported to have a potential role as syntrophic acetate oxidizing bacteria (SAOB) in elevated ammonia concentration [70–72]. It is even more likely to have an active syntrophic acetate oxidation (SAO) associated with *Syntrophomonadaceae* and *Dethiobacteraceae* when the concentration of hydrogenotrophic methanogens is high [73]. The family members of *Dethiobacteraceae* can contribute to a complete ß-oxidation pathway, potentially including fatty acid decomposition in the very initial inoculum which was adapted for fat- and protein-rich substrates [61]. The high relative abundance of *Dethiobacteraceae* and *Methanobacterium* in the initial inoculum and ammonia saturated samples on day 6 may suggest that the SAO was initially the pathway for methane production. In contrast, at the end of the experiment, a shift in the methane production pathway occurred in the system and acetoclastic methanogenesis became the dominant pathway for degradation of lignocellulosic materials. *Guggenheimella* is also a mesophilic fermenter capable of hydrolysis of organic compounds, including petroleum hydrocarbons to propionate, butyrate and acetate as well as small amount of isovalerate, isobutyrate [74–76].

After 120 days of adaptation, the relative abundance of different bacterial families changed as a function of change in OLR as well as the nature of the feedstocks. Comparing the relative abundance of different microbial families in the initial inoculum and samples from day 120 in R_0_ and R_1_, the relative abundance of family members in phylum including *Actinobacteriota *(*Actinomycetaceae*, *Microbacteriaceae and Atopobiaceae*),* Fibrobacterota *(*Fibrobacteraceae*),* Firmicutes *(*Ruminococcaceae*, *Sedimentibacteraceae*, *Caldicoprobacter*), *Cloacimonadota *(*Cloacimonadaceae*), and *Bacteroidota *(*Rikenellaceae*, *Bacteroidales_UCG-001 and GZKB124*) increased significantly (Fig. 5). The relative abundance of *Actinomycetaceae* and *Fibrobacteraceae* were increased by over 310- and 61-fold, respectively. At the same time, the *Methanosarcina* genera represented over 70 and 64% of the archaea in R_0_ and R_1_, respectively. Family members of *Actinobacteriota* are abundant in lignin and fiber-rich environments. These families can degrade complex plant materials and recalcitrant polymers including cellulose and hemicellulose converting them to monosaccharides and volatile acids [77–79]. *Fibrobacteraceae* can be found in the cattle rumen that contribute in complete degradation of cellulosic materials through generation of cellulases enzyme [80]. *Actinobacteriota* and *Fibrobacterota* phyla were not highly abundant in the initial inoculum, whereas they increased in R_0_ and R_1_ by increasing the OLR. Hence, these families could play a joint role as active plant degraders, and moreover, this result indicates that these bacteria are essential in biogas reactors operated at high cellulose loadings*.*

*Sedimentibacter* (*Sedimentibacteraceae*) (17–25%), *Hungateiclostridiaceae* (15–25%), *Ruminococcaceae* (9%) and *Caldicoprobacter* (9%) were the most abundant bacterial families within *Firmicutes* phylum after 120 days. Relative abundance of *Sedimentibacteraceae* was substantially increased in R_1_ and R_0_ on day 120 when the abundancy of *Methanosarcina* was maximum. *Sedimentibacter* are obligate anaerobe capable of direct electron transfer to electrophic methanogens (e.g., *Methanosarcina* and *Methanothrix*) through biological electron wires (also known as e-pili) [81, 82]. Considering a higher biomethane production rate in a constant OLR from day 90 and simultaneous increase in abundance of *Sedimentibacteraceae* and *Methanosarcina* may suggest possibility of direct interspecies electron transfer process. *Ruminococcaceae* are normally isolated from rumen and can contribute in hydrolysis of cellulosic biomass through degrading d-galactitol and glutamate to generate VFAs and hydrogen [83–85]. An increased relevant abundance of *Ruminococcaceae* during the microbial adaptation process can be linked to reduced crystallinity of cellulosic materials as a result of steam explosion. *Caldicoprobacter* species belonging to Clostridia are thermophilic anaerobic bacteria capable of converting carbohydrates (e.g., xylan) [86], amino acids and VFAs specially (propionate) to acetate and CO_2_. Dyksma et al. also suggested that in harsh operation conditions (e.g., elevated temperature and pH) *Caldicoprobacter* may contribute in acetate oxidation route together with hydrogenotrophic methanogens [61]. Presence of *Caldicoprobacter* in this mesophilic condition might be associated with a favorable condition for their growth including high concentration of xylan (from hemicellulose degradation during steam explosion) and propionic acid in elevated pH and ammonia level. Moreover, developing a syntrophic partnership with hydrogen consumers can also be linked to high relative abundance of *Caldicoprobacter* [87]. The relative abundance of *Caldicoprobacter* multiplied significantly in early days of adaptations (e.g., day 6 and day 35) which can be related to *Caldicoprobacter*’s abilities to degrade hemicellulose. Compared to cellulose and cellulosic polysaccharides with crystalline structure, hemicellulose is easier to be degraded. Consequently, relative abundance of *Caldicoprobacter* increased faster in comparison with cellulose hydrolysis bacteria [24, 88].

Members of *Cloacimonadota* (previously Cloacimonetes, WWE1) family W27 are involved in degradation of long-chained fatty acids in fat-rich cultures through development of syntrophic ß-oxidation [89, 90]. Considering the nature of initial inoculum’s feedstock (fat and protein), it may explain the high relative abundance of family W27 belonging to *Cloacimonadota* at the early days of the experiment, but it was not observed after day 35 in R0 and R1. In contrast, the relative abundance of *Cloacimonadaceae* families within *Cloacimonadota* phylum increased as a factor of OLR of SEBW. The *Cloacimonadaceae* W5 is suggested to be a syntrophic propionate oxidizing bacteria in anaerobic culture [91]. A high concentration of propionic acid (Table 2) in the samples during the adaptation period may have developed a suitable condition for the growth of *Cloacimonadaceae* W5. *Bacteroidota* can generate a wide range of extracellular hydrolytic enzymes converting glucose, arabinose, cellobiose and other carbohydrates in lignocellulosic biomass to acetic acid, butyric acid, isovaleric acid, Propionic, H_2_ and CO_2_ [92]. An increase in the relevant abundance of *Rikenellaceae*, *Bacteroidales*_UCG-001 and GZKB124 belonging to *Bacteroidota* phylum as a result of a long-term operation with SEBW and CM reveal that the microbial community adaptation improved hydrolysis efficiency [77, 89].

### Stable carbon isotope analysis

Combined relative abundance of hydrogenotrophic methanogens (i.e., *Methanobacterium*, *Ca_Methanofastidiosum*, *Methanobrevibacter*, *Methanocorpusculum*, and *Methanoculleus*) and acetoclastic methanogens (i.e., *Methanosarcina* and *Methanothrix*) at each sampling point have been presented in Table 3. Apparent fraction factors of each sampling point were calculated according to a method described in “Measurement of the stable isotope composition of the biogas”. Moreover, the concentration of ammonium and VFAs and pH measurements are provided in Table 3. The total abundance of hydrogenotrophic archaea on day 0 was around 86%. On the same sampling day, the α_C_ for R_0_ and R_1_ was 1.054 and 1.050, respectively. On day 6, when the pH increased to over 7.9 in both reactors, the concentration of hydrogenotrophic methanogens increased significantly and reached around 99 percent. On the same day, the α_C_ also rose and ran to its maximum of 1.058 and 1.062 in R_0_ and R_1_. These results were in line with the results of the literature where the α_C_ close to or over 1.06 represents a dominant hydrogenotrophic methanogenesis pathway [35]. From day 6 on, when feeding the reactors with SEBW and CM was initiated, the VFA concentration in the reactors increased, reaching a maximum of 1.1 and 2.7 g/L in R_0_ and R_1_ on day 120, respectively. Similarly, on day 120, the relevant abundance of acetoclastic methanogens (more precisely *Methanosarcina*) extended to 65.5 and 73.4% in R_0_ and R_1_, respectively. In contrast, apparent fraction factors in all samples decreased by 1.024 and 1.027 in R_0_ and R_1_, respectively.

**Table 3:** Relative abundance of methane-producing archaea and the apparent fraction factors *α*_c_

	Hydrogenotrophic methanogens	Acetoclastic methanogens	*α*_c_	NH_4_	VFA	pH
R_0_						
Day 0	85.9	14.1	1.054	1.9	0.4	7.52
Day 6	98.9	1.1	1.058	2.8	0.8	7.92
Day 35	56.8	43.2	1.04	1.05	0.5	7.47
Day 65	39.1	60.9	1.03	0.6	1.07	7.36
Day 95	36.4	63.6	1.025	0.6	1.01	7.38
Day 120	34.5	65.5	1.024	0.57	1.1	7.38
R_1_						
Day 0	85.8	14.2	1.05	1.7	0.2	7.51
Day 6	99.19	0.9	1.062	3	1.01	7.91
Day 35	49.6	50.4	1.04	1.1	0.5	7.45
Day 65	33.2	66.8	1.035	0.6	1.7	7.35
Day 95	26.3	73.7	1.032	0.54	1.6	7.35
Day 120	26.6	73.4	1.027	0.48	2.7	6.97

Presented ammonium, VFA and pH are the average value of three parallel measurements from the same samples

**Fig. 6:**
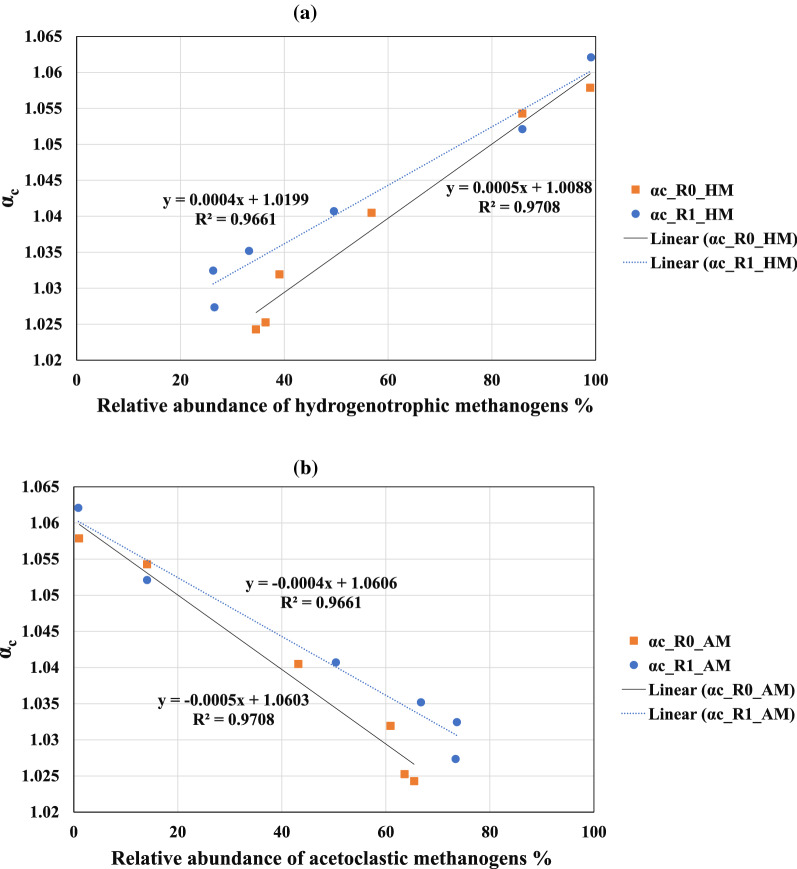
Correlation between apparent carbon isotope fraction (αc) and relative abundance of hydrogenotrophic methanogens (**a**) or relative abundance of acetoclastic methanogens (**b**) at six sampling points

Stable carbon isotope signature of biogas samples from the initial inoculum and day 6, along with the results from the genetic analysis, revealed that the acetate oxidation pathway through potential syntrophic bacteria (e.g., *Cloacimonadota*, *Dethiobacteraceae*, and *Syntrophomonadaceae*) and hydrogenotrophic methanogens (specially *Methanobacterium* and *Methanoculleus*) could have a major contribution in methane production at the very beginning days of the experiment. After a long period of adaptation, the relative abundance of syntrophic bacteria diminished, yet did not totally disappear, leading to a tangible shift in the methane production pathways where acetoclastic methanogen became the dominant methane-producing pathway. As shown in Fig. 6, the calculated apparent fraction factor (*α*_C_) in this study is well correlated to the abundance of the methanogens meaning that the dominant methane-producing pathway in the system can be estimated using the stable ^13^C isotope of the biogas.

The change in the carbon isotope fractionation factor was well correlated to the dominating methanogenic group. The fraction of methane formed from reduction of CO_2_ was clearly reduced as the relative abundance of acetoclastic methanogens increased. Similar correlations have previously been reported, and e.g., Vavilin and Ritov found that at various ammonium concentrations, when acetoclastic methanogenesis dominated, the fractionation factor value decreases over time to a low level of 1.016 [93]. Under conditions were hydrogenotrophic methanogenesis was dominating the value increased to 1.060. These results are in accordance with the pattern observed in R_0_ and R_1_; however, the lowest fractionation found in our study were 1.024 and 1.027. This is somewhat higher than the levels found in the literature indicating that that methane also partly was produced via acetate oxidation and CO_2_.

The high fraction factor found for the initial inoculum (close to 1.060) implies that the source reactor was highly dominated by hydrogenotrophic methanogenesis. *Methanosaeta* normally plays an important role in acetoclastic methanogenesis, and typically competes with *Methanosarcina* for acetate. *Methanosaeta* is reported to dominate at low ammonium concentrations, while *Methanosarcina* dominates at the higher ammonium concentrations [93]. Methanosarcina will also typically dominate when acetate concentrations are high [94]. The ammonium levels in the initial inoculums (day 0) and early stage (day 6) of experiments where relatively high (Table 3), and this together with increasing concentration of VFA (acetic acid) can explain why *Methanosarcina* was the dominating methanogen.

In both reactors, at day 6, the ammonium concentrations were at its highest and were decreasing for the rest of the experiment. At this measuring point, also the fractionation factor was at the maximum level. This implies that hydrogenotrophic methanogenesis increased during the start-up, which can be explained by the high amount of initial inoculum in the reactors during that period and also that the microbes were not yet adapted to the new conditions, and the process was unstable (which is also supported by the results for biogas production, decreasing between day 5 and 15). It is reported that hydrogenotrophic methanogenic communities are more robust and capable of maintenance in stressful conditions, which is the probable explanation for the pattern observed in R_0_ and R_1_. Moreover, these data show that the changes in microbial methane pathway can change relatively rapidly as a response to shifts in the environment.

## Conclusions

Biomethane production from SEBW after a long adaptation period yielded around 70% of the theoretical methane production yield of SEBW–CM mixture. It was also shown that the adaptation could increase the microbial community’s tolerance threshold against inhibitors (i.e., furfural and HMF) from less than 1 g/L to 2 g/L. Both the microbial community and the fractionated stable isotopes analyses showed a clear shift from hydrogenotrophic to acetoclastic methanogenesis as the culture was adapted to the lignocellulosic feedstock, and the results suggest that direct interspecies electron transfer could be involved in the increased methane production in R0 with constant OLR. During the adaptation period, the bacterial community composition changed from SOA-rich culture to cellulose/hemicellulose hydrolyzer culture, and the methane production pathway shifted from hydrogenotrophic to the acetoclastic path. This shows that the hydrolysis step is important in the AD of lignocellulosic materials. Altogether, the findings in this study provide an increased understanding of feedstock composition and reactors operations impact on the microbial dynamics and isotope fractionation of biogas from AD. Moreover, the tight correlations between shifts in microbial composition and stable isotopes may improve the biogas plants’ condition to monitor changes in the process and put in place measures to prevent instability and process collapse.

## Supplementary Information

Additional file 1. Microbial community analysis

## Data Availability

The authors declare that this is our original work, and the corresponding manuscript has not been published before and is only submitted to the Journal of Biotechnology for Biofuels and Bioproducts. The supporting biological data and analysis have been provided as Additional file 1 that can be available both for reviewers and for the readers of the journal. Seyedbehnam Hashemi, as the corresponding author, confirms that the submission of this manuscript has been approved by all the above-mentioned authors.

## References

[1] International Energy Agency. Net zero by 2050—a roadmap for the global energy sector. 2021.

[2] Obersteiner M, Azar Ch, Kauppi P, Möllersten K, Moreira J, Nilsson S (2001). Managing climate risk. Science (1979).

[3] Fuss S, Reuter WH, Szolgayová J, Obersteiner M (2013). Optimal mitigation strategies with negative emission technologies and carbon sinks under uncertainty. Clim Change.

[4] Raut MP, Pandhal J, Wright PC (2021). Effective pretreatment of lignocellulosic co-substrates using barley straw-adapted microbial consortia to enhanced biomethanation by anaerobic digestion. Bioresour Technol.

[5] Kabir MM, Forgács G, Horváth IS (2015). Biogas from lignocellulosic materials.

[6] Hashemi B, Sarker S, Lamb JJ, Lien KM (2021). Yield improvements in anaerobic digestion of lignocellulosic feedstocks. J Clean Prod.

[7] Ravindran R, Jaiswal AK (2016). A comprehensive review on pre-treatment strategy for lignocellulosic food industry waste: challenges and opportunities. Bioresour Technol.

[8] Paul S, Dutta A (2018). Challenges and opportunities of lignocellulosic biomass for anaerobic digestion. Resour Conserv Recycl.

[9] Shrestha S, Fonoll X, Khanal SK, Raskin L (2017). Biological strategies for enhanced hydrolysis of lignocellulosic biomass during anaerobic digestion: current status and future perspectives. Bioresour Technol.

[10] Ziemiński K, Romanowska I, Kowalska M (2012). Enzymatic pretreatment of lignocellulosic wastes to improve biogas production. Waste Manag.

[11] You Z, Pan SY, Sun N, Kim H, Chiang PC (2019). Enhanced corn-stover fermentation for biogas production by NaOH pretreatment with CaO additive and ultrasound. J Clean Prod.

[12] Ilanidis D, Wu G, Stagge S, Martín C, Jönsson LJ (2021). Effects of redox environment on hydrothermal pretreatment of lignocellulosic biomass under acidic conditions. Bioresour Technol.

[13] Khan MU, Ahring BK (2021). Anaerobic biodegradation of wheat straw lignin: the influence of wet explosion pretreatment. Energies.

[14] Shi Q, Li Y, Li Y, Cheng Y, Zhu W (2019). Effects of steam explosion on lignocellulosic degradation of, and methane production from, corn stover by a co-cultured anaerobic fungus and methanogen. Bioresour Technol.

[15] Hashemi S, Joseph P, Mialon A, Moe S, Lamb JJ, Lien KM (2021). Enzymatic pretreatment of steam-exploded birch wood for increased biogas production and lignin degradation. Bioresour Technol Rep.

[16] Horn SJ, Nguyen QD, Westereng B, Nilsen PJ, Eijsink VGH (2011). Screening of steam explosion conditions for glucose production from non-impregnated wheat straw. Biomass Bioenergy.

[17] Yu Y, Wu J, Ren X, Lau A, Rezaei H, Takada M (2022). Steam explosion of lignocellulosic biomass for multiple advanced bioenergy processes: a review. Renew Sustain Energy Rev.

[18] Horn SJ, Estevez MM, Nielsen HK, Linjordet R, Eijsink VGH (2011). Biogas production and saccharification of Salix pretreated at different steam explosion conditions. Bioresour Technol.

[19] Aarum I, Devle H, Ekeberg D, Horn SJ, Stenstrøm Y (2018). Characterization of pseudo-lignin from steam exploded birch. ACS Omega.

[20] Svensson K, Kjørlaug O, Higgins MJ, Linjordet R, Horn SJ (2018). Post-anaerobic digestion thermal hydrolysis of sewage sludge and food waste: effect on methane yields, dewaterability and solids reduction. Water Res.

[21] Pokój T, Klimiuk E, Bułkowska K, Kowal P, Ciesielski S (2020). Effect of individual components of lignocellulosic biomass on methane production and methanogen community structure. Waste Biomass Valoriz.

[22] Neshat SA, Mohammadi M, Najafpour GD, Lahijani P (2017). Anaerobic co-digestion of animal manures and lignocellulosic residues as a potent approach for sustainable biogas production. Renew Sustain Energy Rev.

[23] Conrad R (2020). Methane production in soil environments—anaerobic biogeochemistry and microbial life between flooding and desiccation. Microorganisms.

[24] Hashemi S, Hashemi SE, Lien KM, Lamb JJ (2021). Molecular microbial community analysis as an analysis tool for optimal biogas production. Microorganisms.

[25] Bolado-Rodríguez S, Toquero C, Martín-Juárez J, Travaini R, García-Encina PA (2016). Effect of thermal, acid, alkaline and alkaline-peroxide pretreatments on the biochemical methane potential and kinetics of the anaerobic digestion of wheat straw and sugarcane bagasse. Bioresour Technol.

[26] Fotidis IA, Karakashev D, Kotsopoulos TA, Martzopoulos GG, Angelidaki I (2013). Effect of ammonium and acetate on methanogenic pathway and methanogenic community composition. FEMS Microbiol Ecol.

[27] Desmond-Le Quéméner E, Moscoviz R, Bernet N, Marcus A (2021). Modeling of interspecies electron transfer in anaerobic microbial communities. Curr Opin Biotechnol.

[28] Briones A, Raskin L (2003). Diversity and dynamics of microbial communities in engineered environments and their implications for process stability. Curr Opin Biotechnol.

[29] Nikolausz M, Walter RFH, Sträuber H, Liebetrau J, Schmidt T, Kleinsteuber S (2013). Evaluation of stable isotope fingerprinting techniques for the assessment of the predominant methanogenic pathways in anaerobic digesters. Appl Microbiol Biotechnol.

[30] Bremges A, Maus I, Belmann P, Eikmeyer F, Winkler A, Albersmeier A (2015). Deeply sequenced metagenome and metatranscriptome of a biogas-producing microbial community from an agricultural production-scale biogas plant. Gigascience.

[31] Laukenmann S, Polag D, Heuwinkel H, Greule M, Gronauer A, Lelieveld J (2010). Identification of methanogenic pathways in anaerobic digesters using stable carbon isotopes. Eng Life Sci.

[32] Ito T, Yoshiguchi K, Ariesyady HD, Okabe S (2012). Identification and quantification of key microbial trophic groups of methanogenic glucose degradation in an anaerobic digester sludge. Bioresour Technol.

[33] Lv Z, Chen Z, Chen X, Liang J, Jiang J, Loake GJ (2019). Effects of various feedstocks on isotope fractionation of biogas and microbial community structure during anaerobic digestion. Waste Manage.

[34] Whiticar MJ, Faber E, Schoell M, Whiticar MJ, Faber E, Schoell M (1986). Biogenic methane formation in marine and freshwater environments: CO_2_ reduction vs. acetate fermentation—Isotope evidence. GeCoA.

[35] Lv Z, Liang J, Chen X, Chen Z, Jiang J, Loake GJ (2019). Assessment of the start-up process of anaerobic digestion utilizing swine manure:13c fractionation of biogas and microbial dynamics. Environ Sci Pollut Res.

[36] Horn SJ, Eijsink VGH (2010). Enzymatic hydrolysis of steam-exploded hardwood using short processing times. Biosci Biotechnol Biochem.

[37] Østgaard K, Kowarz V, Shuai W, Henry IA, Sposob M, Haugen HH (2017). Syringe test screening of microbial gas production activity: cases denitrification and biogas formation. J Microbiol Methods.

[38] Albertsen M, Karst SM, Ziegler AS, Kirkegaard RH, Nielsen PH (2015). Back to basics—the influence of DNA extraction and primer choice on phylogenetic analysis of activated sludge communities. PLoS ONE.

[39] Illumina I (2015). 16S metagenomic sequencing library preparation, part# 15044223. Rev B.

[40] Apprill A, McNally S, Parsons R, Weber L (2015). Minor revision to V4 region SSU rRNA 806R gene primer greatly increases detection of SAR11 bacterioplankton. Aquat Microb Ecol.

[41] Sluiter A, Hames B, Ruiz R, Scarlata C, Sluiter J, Templeton D, Crocker D. Determination of structural carbohydrates and lignin in biomass: laboratory analytical procedure (LAP). Technical Report NREL/ TP-510-42618. 2008.

[42] Siegert I, Banks C (2005). The effect of volatile fatty acid additions on the anaerobic digestion of cellulose and glucose in batch reactors. Process Biochem.

[43] Ziels RM, Karlsson A, Beck DAC, Ejlertsson J, Yekta SS, Bjorn A (2016). Microbial community adaptation influences long-chain fatty acid conversion during anaerobic codigestion of fats, oils, and grease with municipal sludge. Water Res.

[44] Seadi T, Rutz D, Janssen R, Drosg B (2013). Biomass resources for biogas production.

[45] Estevez MM, Sapci Z, Linjordet R, Schnürer A, Morken J (2014). Semi-continuous anaerobic co-digestion of cow manure and steam-exploded Salix with recirculation of liquid digestate. J Environ Manage.

[46] Estevez MM, Linjordet R, Morken J (2012). Effects of steam explosion and co-digestion in the methane production from Salix by mesophilic batch assays. Bioresour Technol.

[47] Boopathy R (1996). Methanogenic transformation of methylfurfural compounds to furfural. Appl Environ Microbiol.

[48] Belay N, Boopathy R, Voskuilen G (1997). Anaerobic transformation of furfural by Methanococcus deltae (Delta)LH. Appl Environ Microbiol.

[49] Akobi C, Hafez H, Nakhla G (2016). The impact of furfural concentrations and substrate-to-biomass ratios on biological hydrogen production from synthetic lignocellulosic hydrolysate using mesophilic anaerobic digester sludge. Bioresour Technol.

[50] Rivard CJ, Grohmann K (1991). Degradation of furfural (2- furaldehyde) to methane and carbon dioxide by an anaerobic consortium. Appl Biochem Biotechnol.

[51] Cao GL, Ren NQ, Wang AJ, Guo WQ, Xu JF, Liu BF (2010). Effect of lignocellulose-derived inhibitors on growth and hydrogen production by *Thermoanaerobacterium thermosaccharolyticum* W16. Int J Hydrogen Energy.

[52] Siqueira MR, Reginatto V (2015). Inhibition of fermentative H2 production by hydrolysis byproducts of lignocellulosic substrates. Renew Energy.

[53] Anburajan P, Pugazhendhi A, Park JH, Sivagurunathan P, Kumar G, Kim SH (2018). Effect of 5-hydroxymethylfurfural (5-HMF) on high-rate continuous biohydrogen production from galactose. Bioresour Technol.

[54] Muñoz-Páez KM, Alvarado-Michi EL, Buitrón G, Valdez-Vazquez I (2019). Distinct effects of furfural, hydroxymethylfurfural and its mixtures on dark fermentation hydrogen production and microbial structure of a mixed culture. Int J Hydrogen Energy.

[55] Pekarová S, Dvorácková M, Stloukal P, Ingr M, Šerá J, Koutny M (2017). Quantitation of the inhibition effect of model compounds representing plant biomass degradation products on methane production. BioResources.

[56] Tan Z, Li X, Yang C, Liu H, Cheng JJ (2021). Inhibition and disinhibition of 5-hydroxymethylfurfural in anaerobic fermentation: a review. Chem Eng J.

[57] Park JH, Yoon JJ, Park HD, Lim DJ, Kim SH (2012). Anaerobic digestibility of algal bioethanol residue. Bioresour Technol.

[58] Sundberg C, Al-Soud WA, Larsson M, Alm E, Yekta SS, Svensson BH (2013). 454 pyrosequencing analyses of bacterial and archaeal richness in 21 full-scale biogas digesters. FEMS Microbiol Ecol.

[59] Li A, Chu Y, Wang X, Ren L, Yu J, Liu X (2013). A pyrosequencing-based metagenomic study of methane-producing microbial community in solid-state biogas reactor. Biotechnol Biofuels.

[60] Klocke M, Mähnert P, Mundt K, Souidi K, Linke B (2007). Microbial community analysis of a biogas-producing completely stirred tank reactor fed continuously with fodder beet silage as mono-substrate. Syst Appl Microbiol.

[61] Dyksma S, Jansen L, Gallert C (2020). Syntrophic acetate oxidation replaces acetoclastic methanogenesis during thermophilic digestion of biowaste. Microbiome.

[62] Müller B, Sun L, Westerholm M, Schnürer A (2016). Bacterial community composition and fhs profiles of low- and high-ammonia biogas digesters reveal novel syntrophic acetate-oxidising bacteria. Biotechnol Biofuels.

[63] Mayumi D, Mochimaru H, Yoshioka H, Sakata S, Maeda H, Miyagawa Y (2011). Evidence for syntrophic acetate oxidation coupled to hydrogenotrophic methanogenesis in the high-temperature petroleum reservoir of Yabase oil field (Japan). Environ Microbiol.

[64] Calusinska M, Goux X, Fossépré M, Muller EEL, Wilmes P, Delfosse P (2018). A year of monitoring 20 mesophilic full-scale bioreactors reveals the existence of stable but different core microbiomes in bio-waste and wastewater anaerobic digestion systems. Biotechnol Biofuels.

[65] Heitkamp K, Latorre-Pérez A, Nefigmann S, Gimeno-Valero H, Vilanova C, Jahmad E (2021). Monitoring of seven industrial anaerobic digesters supplied with biochar. Biotechnol Biofuels.

[66] Ravachol J, Borne R, Meynial-Salles I, Soucaille P, Pagès S, Tardif C (2015). Combining free and aggregated cellulolytic systems in the cellulosome-producing bacterium *Ruminiclostridium cellulolyticum*. Biotechnol Biofuels.

[67] Kang YR, Su Y, Wang J, Chu YX, Tian G, He R (2021). Effects of different pretreatment methods on biogas production and microbial community in anaerobic digestion of wheat straw. Environ Sci Pollut Res.

[68] Chen S, Dong B, Dai X, Wang H, Li N, Yang D (2019). Effects of thermal hydrolysis on the metabolism of amino acids in sewage sludge in anaerobic digestion. Waste Manag.

[69] Poirier S, Déjean S, Midoux C, Lê Cao KA, Chapleur O (2020). Integrating independent microbial studies to build predictive models of anaerobic digestion inhibition by ammonia and phenol. Bioresour Technol.

[70] Zhilina TN, Zavarzina DG, Kolganova TV, Tourova TP, Zavarzin GA (2005). “Candidatus Contubernalis alkalaceticum”, an obligately syntrophic alkaliphilic bacterium capable of anaerobic acetate oxidation in a coculture with desulfonatronum cooperativum. Microbiology.

[71] Sorokin DY, Abbas B, Geleijnse M, Kolganova TV, Kleerebezem R, van Loosdrecht MCM (2016). Syntrophic associations from hypersaline soda lakes converting organic acids and alcohols to methane at extremely haloalkaline conditions. Environ Microbiol.

[72] Liu P, Qiu Q, Lu Y (2011). Syntrophomonadaceae-affiliated species as active butyrate-utilizing syntrophs in paddy field soil. Appl Environ Microbiol.

[73] Li C, He P, Hao L, Lü F, Shao L, Zhang H (2022). Diverse acetate-oxidizing syntrophs contributing to biogas production from food waste in full-scale anaerobic digesters in China. Renew Energy.

[74] Tu YT, Chiang PC, Yang J, Chen SH, Kao CM (2014). Application of a constructed wetland system for polluted stream remediation. J Hydrol.

[75] Zhang L, Ban Q, Li J, Zhang S (2022). An enhanced excess sludge fermentation process by anthraquinone-2-sulfonate as electron shuttles for the biorefinery of zero-carbon hydrogen. Environ Res.

[76] Wyss C, Dewhirst FE, Paster BJ, Thurnheer T, Luginbühl A (2005). *Guggenheimella bovis* gen. nov., sp. nov., isolated from lesions of bovine dermatitis digitalis. Int J Syst Evol Microbiol.

[77] Zhong Y, He J, Zhang P, Zou X, Pan X, Zhang J (2022). Effects of different particle size of zero-valent iron (ZVI) during anaerobic digestion: performance and mechanism from genetic level. Chem Eng J.

[78] Mirmohamadsadeghi S, Karimi K, Azarbaijani R, Parsa Yeganeh L, Angelidaki I, Nizami AS (2021). Pretreatment of lignocelluloses for enhanced biogas production: a review on influencing mechanisms and the importance of microbial diversity. Renew Sustain Energy Rev.

[79] Li BY, Xia ZY, Gou M, Sun ZY, Huang YL, Jiao SB (2022). Production of volatile fatty acid from fruit waste by anaerobic digestion at high organic loading rates: performance and microbial community characteristics. Bioresour Technol.

[80] Qi M, Nelson KE, Daugherty SC, Nelson WC, Hance IR, Morrison M (2005). Novel molecular features of the fibrolytic intestinal bacterium *Fibrobacter intestinalis* not shared with *Fibrobacter succinogenes* as determined by suppressive subtractive hybridization. J Bacteriol.

[81] Feng D, Xia A, Huang Y, Zhu X, Zhu X, Liao Q (2022). Effects of carbon cloth on anaerobic digestion of high concentration organic wastewater under various mixing conditions. J Hazard Mater.

[82] Zhao Z, Wang J, Li Y, Zhu T, Yu Q, Wang T (2020). Why do DIETers like drinking: metagenomic analysis for methane and energy metabolism during anaerobic digestion with ethanol. Water Res.

[83] Wojcieszak M, Pyzik A, Poszytek K, Krawczyk PS, Sobczak A, Lipinski L (2017). Adaptation of methanogenic inocula to anaerobic digestion of maize silage. Front Microbiol.

[84] Liu Y, Wachemo AC, Yuan HR, Li XJ (2019). Anaerobic digestion performance and microbial community structure of corn stover in three-stage continuously stirred tank reactors. Bioresour Technol.

[85] Zhai S, Li M, Xiong Y, Wang D, Fu S (2020). Dual resource utilization for tannery sludge: effects of sludge biochars (BCs) on volatile fatty acids (VFAs) production from sludge anaerobic digestion. Bioresour Technol.

[86] Jensen MB, de Jonge N, Dolriis MD, Kragelund C, Fischer CH, Eskesen MR (2021). Cellulolytic and xylanolytic microbial communities associated with lignocellulose-rich wheat straw degradation in anaerobic digestion. Front Microbiol.

[87] Bouanane-Darenfed A, Ben HW, Hacene H, Cayol JL, Ollivier B, Fardeau ML (2013). *Caldicoprobacter guelmensis* sp. nov., a thermophilic, anaerobic, xylanolytic bacterium isolated from a hot spring. Int J Syst Evol Microbiol.

[88] Wang Y, Zhang J, Sun Y, Yu J, Zheng Z, Li S (2020). Effects of intermittent mixing mode on solid state anaerobic digestion of agricultural wastes. Chemosphere.

[89] Perman E, Schnürer A, Björn A, Moestedt J (2022). Serial anaerobic digestion improves protein degradation and biogas production from mixed food waste. Biomass Bioenergy.

[90] Shakeri Yekta S, Liu T, Axelsson Bjerg M, Šafarič L, Karlsson A, Björn A (2019). Sulfide level in municipal sludge digesters affects microbial community response to long-chain fatty acid loads. Biotechnol Biofuels.

[91] Dyksma S, Gallert C (2022). Effect of magnetite addition on transcriptional profiles of syntrophic *Bacteria* and *Archaea* during anaerobic digestion of propionate in wastewater sludge. Environ Microbiol Rep.

[92] Ziganshina EE, Belostotskiy DE, Bulynina SS, Ziganshin AM (2021). Effect of magnetite on anaerobic digestion of distillers grains and beet pulp: operation of reactors and microbial community dynamics. J Biosci Bioeng.

[93] Vavilin VA, Rytov SV (2017). Dynamic changes of carbon isotope apparent fractionation factor to describe transition to syntrophic acetate oxidation during cellulose and acetate methanization. Isotopes Environ Health Stud.

[94] Conklin A, Stensel HD, Ferguson J (2006). Growth kinetics and competition between methanosarcina and methanosaeta in mesophilic anaerobic digestion. Water Environ Res.

